# The chemical quality of frozen Vietnamese *Pangasius hypophthalmus* fillets

**DOI:** 10.1002/fsn3.302

**Published:** 2015-10-26

**Authors:** Carlos Frederico Marques Guimarães, Eliane Teixeira Mársico, Maria Lúcia Guerra Monteiro, Môsar Lemos, Sergio Borges Mano, Carlos Adam Conte Junior

**Affiliations:** ^1^ Departamento de Tecnologia de AlimentosUniversidade Federal Fluminense24230340NiteróiRio de JaneiroBrazil

**Keywords:** DMA‐80, fraud, freshwater fish, HPLC, mercury, sutchi catfish

## Abstract

The aim of the present study was to evaluate the chemical quality parameters regarding frozen *Pangasius hypophthalmus* specimens from Vietnam. The proximate composition, pH, ammonia, biogenic amines (BAs), total mercury (Hg), malondialdehyde (MDA), and polyphosphate were determined. The moisture, protein, lipid and ash values were between 83.83–85.59, 12.51–14.52, 1.09–1.65, and 0.76–2.38 g 100 g^−1^, respectively. Fraud by excessive polyphosphate addition was detected in 30% of the samples whereas Hg above the recommended limit was observed in 50% of the samples. With regard to compounds from the degradation process, low concentrations of individual BAs and pH values were found in this study and ranged from 5.88 to 6.18, except for samples with polyphosphate >1 g 100^−1^ (pH > 7.00) were observed in the present study. However, ammonia concentration indicated that a degradation process initiated in 80% of the samples (0.12–0.34 NH_3_ g^−1^) and 20% of the samples (1.87–1.94 *μ*g NH_3_ g^−1^) were in an advanced deterioration process. Furthermore, MDA values (1.21–7.88 mg kg^−1^) suggested some failures, mainly during transportation and/or storage. We concluded that quality control measures must be implemented on the *Pangasius* production chain to improve the quality of products provided to consumers worldwide.

## Introduction

World aquaculture production has grown steadily in recent years. In 2012, the aquaculture production increased 12% in relation to 2011 (from an average of 59.0 million tons in 2011 to 66.6 million tons in 2012) (FAO 2014). Among farmed fish species, *Pangasius hypophthalmus* is mainly produced in Asian countries such as Bangladesh, Vietnam, Malaysia, Indonesia, Laos, Cambodia and China. Vietnam is the largest Pangas‐producing country (Phan et al. 2009). This fish species is also known as *Pangasianodon hypophthalmus*, sutchi catfish, striped catfish or tra fish and has been widely exported due to great acceptability, affordable cost, and white meat, which can replace cod or other more expensive white fishes. Currently, sutchi catfish fillets have been exported to over 80 countries worldwide including Netherlands, Germany, and United States, which demand mainly frozen fillets without skin and bone (Phan et al. 2009; Karl et al. 2010). In 2011, striped catfish was ranked the sixth favorite fish species in the United States (FAO 2012). Although Brazil is not an expressive importer of *Pangasius* fillets as Japan and United States of America (FAO 2014), the imported amount of frozen fish fillets from China, Argentina, Chile, and Vietnam increased 117% in 2010 when compared to 2009 (Brazil 2012). Moreover, *Pangasius hypophthalmus* represents a cheaper species imported from Asia and has been commonly sold in Brazil as flounder, a fish species more expensive (Carvalho et al. 2015).

On the other hand, fish is highly perishable, and it is important to evaluate spoilage indicators (quality loss), toxic compound formation and commercial frauds in order to meet the safety standards required by importing countries (Phuong and Oanh 2010). Ammonia is a compound resulting from the bacterial or enzymatic degradation of protein, which increases in fishes during storage and can be used as a spoilage indicator (Debevere and Voets 1973; Rodrigues et al. 2013).

Biogenic amines (BAs) are produced, mainly, by exogenous decarboxylase enzymes (produced by microorganisms) (Shalaby 1996) and may be considered a quality indicator of fish during storage (Cunha et al. 2013; Rodrigues et al. 2013). The ingestion of high levels of BAs may result in severe toxicological effects (Muñoz‐Atienza et al. 2011), including dermatological, gastrointestinal, cardiac and neurological symptoms, and even death (Shalaby 1996; Juneja and Sofos 2010; Muñoz‐Atienza et al. 2011). In addition, BAs may react with nitrates resulting in nitrosamines, which are carcinogenic compounds (Juneja and Sofos 2010).

Malondialdehyde (MDA) is a secondary product from lipid oxidation. This compound in high amounts decreases the paraoxonase (PON1) level. In turn, this leads to a greater risk of dyslipidemia, insulin resistance and high blood pressure, which are considered important components in the pathogenesis of the metabolic syndrome, mainly in obese adolescents (Zaki et al. 2014). In addition, aldehydes have been associated with putative mutagens and cancer formation (Duthie et al. 2013). Regarding environmental pollutants, methyl mercury is the molecular form from mercury (Hg) most detrimental for fish species and humans due to their great toxicity (Minh 2015). Furthermore, methyl mercury exposure as well as high MDA values can be associated with coronary diseases (Yoshizawa et al. 2002; Zaki et al. 2014), emphasizing the need for the implementation of a quality control system.

Polyphosphate is an additive that enhances water binding of fish flesh and is commonly used during the processing of conventionally farmed fish prior to freezing (Carneiro et al. 2013a). Nevertheless, the addition of polyphosphate to deep frozen fish fillets is restricted due to an increase in the water content of thawed fillets and to the remarkable decrease in protein content. This water‐binding additive is considered a commercial fraud when used above the permitted limit (Karl et al. 2010).

In spite of the knowledge about the toxicity of these compounds and the great economic importance of the *Pangasius hypophthalmus*, there is a lack of information available about different chemical quality parameters which are considered quality indicators of fish species. Most of the studies about *Pangasius hypophthalmus* refer to its bacterial quality (Noseda et al. 2012; Tong Thi et al. 2013, 2014; Kulawik et al. 2015; Tong Thi et al. 2015) or individual chemical parameters such as mercury, biogenic amines, and polyphosphate (Orban et al. 2008; Karl et al. 2010; Bunka et al. 2013; Kulawik et al. 2015). Therefore, the aim of this study was to investigate the safety aspects based on several chemical quality parameters of *Pangasius hypophthalmus* fillets from Vietnam.

## Materials and Methods

### Chemicals

All reagents, standards and solvents were purchased from Merk (Darmstadt, Germany), Millipore (Molsheim, France), Sigma‐Aldrich (Saint Louis, MO), and Tedia (Rio de Janeiro, Brazil). All reagents for biogenic amine determination were of HPLC analytical grade.

### Sampling

Ten frozen samples of commercial *Pangasius* fillets were purchased at different supermarkets in Rio de Janeiro, Brazil, and transported to the laboratory. Information regarding fish's origin was obtained from the package label. The time period from purchase to arrival at the laboratory did not exceed 1 h. Before analysis, the samples were thawed at 4°C until they reached room temperature (25°C). Proximate composition, ammonia, BAs, total mercury, polyphosphate, and MDA quantification were assessed. All analyses were made three times.

### Proximate composition

Moisture was determined by using a drying oven at 100–105°C until constant weight, and protein content was estimated by the Kjeldahl method, using 6.25 as the Nitrogen conversion factor. Ash content was determined after incineration in a muffle furnace (550°C), and total lipid content was determined by the Soxhlet extraction method and the petroleum ether solvent in accordance with the Association of Official Analytical Chemists (AOAC 2012).

### Polyphosphate analysis

Polyphosphate concentration was determined through the microcolorimetric method, using ammonium molybdate solution based on the calibration curve with a standard solution of polyphosphate (0.04 mol/L). The homogenized sample (2 g) was put in a crucible, and 2 g of magnesium oxide, and 10 mL of distilled water were added. Then, samples were dried in a hot water bath and carbonized using a Bunsen flame. The crucible was placed in the furnace at 550°C for 3 h and cooled in the desiccator for 30 min. Then, the ash was dissolved with hydrochloric acid solution (1:1, v/v) and filtered into a 250 mL volumetric flask through a qualitative filter with the aid of distilled water until reaching complete volume. An aliquot of 5 mL was transferred into a volumetric flask of 50 mL. One mL of ammonium molybdate solution, 1 mL of hydroquinone, and 1 mL of sodium sulfite were added. The solution was shaken after the addition of each reagent. The volumetric flask was then filled with distilled water and the content was once again shaken followed by a 30‐min rest in the dark. The reading was performed with a SmartSpec^™^ Plus spectrophotometer (Bio‐Rad Laboratories, São Paulo, Brazil) at 650 nm. Results were reported in total concentrations of pyrophosphate and tripolyphosphate (expressed as g 100 g^−1^ P_2_O_5_) (Brazil 1981).

### Ammonia and pH

The quantitative determination of ammonia was adapted according to the colorimetric method of McCullough (1967). Briefly, 10 mL of Milli‐Q water (Millipore, Molsheim, France) was poured into a 15 mL tube containing 1 g of sample. The tube was homogenized for 30 sec and, after decanting, 1 mL of the supernatant was removed. The supernatant was transferred into another tube and 2 mL of Nessler's reagent (Merk, Darmstadt, Germany) was added. Next, the tube was homogenized for 30 sec and measurement was performed immediately with the SmartSpec^™^ Plus spectrophotometer (Bio‐Rad Laboratories) at 425 nm.

Ammonia quantification was determined, using a calibration curve after reading the following seven different concentrations of a standard solution of ammonium sulfate (2 *μ*g /mL) as in the following: 1, 2.5, 5, 7.5, 10, 12.5, and 15 *μ*g NH_3_/g. Each concentration was added to a 15 mL tube containing 1 mL of tungstate. One mL of N sulfuric acid was added and the tube was homogenized (30 sec), and centrifuged at 503 × *g* for 10 min. The protein‐free supernatant (0.5 mL) was removed and transferred into a 15 mL tube. Nessler's reagent (2 mL) was added, the tube was homogenized for 30 sec and the measurement was performed immediately on the SmartSpec^™^ Plus spectrophotometer (Bio‐Rad Laboratories) at 425 nm. Results were expressed in *μ*g NH_3_ g^−1^.

The pH values were measured according to Conte‐Júnior et al. (2008) using a digital pH meter (Digimed^®^ DM‐22) equipped with a DME‐R12 electrode (Digimed^®^).

### Biogenic amines

Biogenic amines (putrescine, cadaverine, histamine, spermine, and spermidine) were determined based on reverse‐phase HPLC. The extraction of biogenic amines was performed according to Lázaro et al. (2013) utilizing a 5% perchloric acid for BAs extraction and 40 *μ*L of benzoyl chloride for derivatization. The chromatographic conditions followed the method described by Baptista et al. (2014). The chromatographic system consisted of an automatic equipment, including a LC20AD pump coupled to a SPDM20A UV–Vis detector and a CBM20A integrator (Shimadzu, Kyoto, Japan). The mobile phase was prepared by mixing acetonitrile and ultrapure water 42:58 (v/v), the injected volume was 20 *μ*L, the flow rate was 1 mL min^−1^, and the total run time was 15 min under isocratic conditions. Moreover, the column temperature was 20°C, and the detector wavelength was set at 198 nm.

### Lipid oxidation

Malondialdehyde values were determined using the distillation method and a 2‐thiobarbituric acid (TBA) according to Tarladgis et al. (1960). The absorbance values were determined utilizing a SmartSpecTM Plus Spectrophotometer (Bio‐Rad Laboratories) at 538 nm against a blank containing 5 mL of distilled water and 5 mL of TBA solution. Results were expressed in mg MDA kg^−1^ after multiplying the absorbance by 7.8

### Mercury analysis

Total Hg was determined using a Direct Mercury Analyzer (DMA‐80, Milestone, Bergamo, Italy) following the manufacturer's recommendations. The sample (0.27 g) was put in a quartz tube and dried in an oxygen stream which was used as combustion and carrier gases. The sample evaporation occurred in three steps as in the following: 160°C for 1 min, 650°C for 2 min, and 650°C for 1 min. Finally, Hg vapor was desorbed for quantification using a gold amalgamation trap. Hg was quantified using a calibration curve (*R* ^2 ^= 0.9990) taking into account sample weight and peak height. Results were expressed in mg kg^−1^.

### Statistical analysis

All fish analyzed in this experiment were considered as total frequency (%, *n* = 10) and were evaluated as dependent variable with regard to other variables such as physicochemical parameters.

## Results and discussion

### Proximate composition

The proximate composition results are presented in Table 1. All samples exhibited moisture, protein, lipid, and ash values between 83.83–85.59, 12.51–14.52, 1.09–1.65, and 0.76–2.38 g 100 g^−1^, respectively. Similar fluctuations were reported by Pongpet et al. (2015) in *Pangasianodon hypophthalmus* and *Pangasius bocourti* fillets and Orban et al. (2008) in *Pangasius hypophthalmus* fillets. Karl et al. (2010) investigated the proximate composition of conventionally farmed *Pangasius* fillets and their results are partially in accordance with the findings of this study, except for lipid content, which varied between 1.4–3.2%.

**Table 1:** Proximate composition of *Pangasius hypophthalmus* fillets from Vietnam marketed in Brazil

Samples	Composition (g 100 g^−1^)			
	Moisture	Protein	Lipid	Ash
1	84.82 ± 0.08	12.51 ± 0.07	1.65 ± 0.04	0.77 ± 0.04
2	83.83 ± 0.06	13.04 ± 0.04	1.30 ± 0.06	1.07 ± 0.07
3	84.17 ± 0.06	13.21 ± 0.07	1.19 ± 0.06	0.96 ± 0.04
4	83.84 ± 0.08	14.52 ± 0.05	1.09 ± 0.03	0.76 ± 0.03
5	84.18 ± 0.08	13.85 ± 0.04	1.30 ± 0.05	0.87 ± 0.04
6	84.06 ± 0.07	13.56 ± 0.05	1.24 ± 0.04	0.76 ± 0.03
7	84.24 ± 0.06	14.10 ± 0.04	1.20 ± 0.05	0.95 ± 0.05
8	85.59 ± 0.06	12.69 ± 0.02	1.32 ± 0.06	2.38 ± 0.06
9	85.05 ± 0.06	13.55 ± 0.06	1.24 ± 0.02	1.11 ± 0.02
10	84.22 ± 0.01	13.94 ± 0.06	1.37 ± 0.04	0.85 ± 0.04

Values were expressed as mean ± standard deviation.

Regarding total lipid content, in general, fishes are divided into groups as follows: lean (<2%), low‐fat (2–4%), medium‐fat (4–8%) and high‐fat (>8%). In addition, fishes with high amounts of protein content must contain greater than 15% (Stansby 1976). Our findings indicate that *Pangasius hypophthalmus* fillets present lipid content equivalent to lean meat and are not considered a high protein fish meat. In line with our results, Islami et al. (2014) reported 1.82% of lipid in *Pangasianodon hypophthalmus*. On the other hand, Domiszewski et al. (2011) demonstrated high lipid values (2.23%), whereas Islami et al. (2014) reported great protein content (15.97%) in *Pangasianodon hypophthalmus*.

Proximate composition of fishes is highly variable mainly due to species, catching season, environment, diet, age, sex (Boran and Karaçam 2011), and the presence of prohibited additives or additives above the recommended limit (Karl et al. 2010). Mushahida‐Al‐Noor et al. (2012) affirmed that different diets result in a wide variation in proximate compositions of *Pangasius hypophthalamus* fillets (74.10–79.15% moisture, 15.50–16.60% protein, 4.08–8.08% lipid, and 1.20–1.24% ash). Furthermore, Karl et al. (2010) reported greater moisture content and lower protein level in *Pangasius hypophthalamus* fillets with added polyphosphate compared to the fillets without any addition, which is in agreement with our results. In general, the same samples (30%) tended to have high moisture and low protein contents.

### Polyphosphate

Polyphosphates represent food additives that are permitted in products due to their freezing capacity and their ability to improve water‐holding capacity and decrease the thaw drip (Carneiro et al. 2013a,b; FDA 2006). However, their excessive use results in economic fraud and undesirable chemical changes in foods. In general, polyphosphate treatment is mainly employed to treat fish, fish fillet, shrimp, and meat products ranging from 2 to 6%, and resulting in approximately 0.5% of residual phosphate (Gonçalves and Ribeiro 2009). The Codex Alimentarius recommends 1 g 100 g^−1^ of phosphates in the final product (Codex Alimentarius 2011). However, its adequate use is also controlled by good manufacturing practices.

There is evidence of the use of water retaining agents in *Pangasius* fish exported to the European Union (Karl et al. 2010). Nevertheless, there is a lack of studies on polyphosphate concentration in fish and fishery products as a control measurement in order to avoid fraud. The results of the present study demonstrated polyphosphate levels between 0–10.74 g 100 g^−1^ (Table 2). Polyphosphate was not detected in only 10% of the samples, whereas 30% of the samples exhibited polyphosphate amounts > 1 g 100 g^−1^, which can be considered fraud. In addition, polyphosphate results explained the higher moisture, the lower protein, and the greater pH values in samples 1, 8, and 9 compared to other samples evaluated. Similar findings were reported by Karl et al. (2010) in *Pangasius* fillets in the identification of samples treated with polyphosphate.

**Table 2:** Physicochemical parameters of *Pangasius hypophthalmus* fillets from Vietnam marketed in Brazil

Samples	MDA	Hg	pH	Ammonia	Polyphosphate
1	1.63 ± 0.01	0.03 ± 0.00	7.93 ± 0.01	0.26 ± 0.02	2.20 ± 0.03
2	1.52 ± 0.02	0.51 ± 0.01	6.00 ± 0.02	0.22 ± 0.01	0.04 ± 0.00
3	1.79 ± 0.01	0.01 ± 0.00	6.15 ± 0.01	0.29 ± 0.02	0.02 ± 0.00
4	1.21 ± 0.02	0.03 ± 0.00	5.91 ± 0.01	0.26 ± 0.01	ND
5	2.53 ± 0.01	0.64 ± 0.01	6.11 ± 0.01	0.32 ± 0.03	0.04 ± 0.00
6	1.93 ± 0.02	0.02 ± 0.00	6.18 ± 0.01	0.34 ± 0.02	0.05 ± 0.00
7	3.55 ± 0.02	1.31 ± 0.00	5.89 ± 0.01	0.29 ± 0.02	0.01 ± 0.00
8	7.44 ± 0.02	0.76 ± 0.00	8.01 ± 0.02	1.87 ± 0.03	10.74 ± 0.02
9	7.88 ± 0.01	0.05 ± 0.00	7.20 ± 0.00	1.94 ± 0.03	4.93 ± 0.02
10	3.58 ± 0.02	0.70 ± 0.01	5.88 ± 0.01	0.12 ± 0.01	0.03 ± 0.00

ND, not detectable.

Values were expressed as mean ± standard deviation.

MDA and Hg results were expressed as mg kg^−1^.

Ammonia values were expressed as *μ*g NH_3_ g^−1^.

Polyphosphate results were expressed as g 100 g^−1^.

Further, we suggest that a quantitative polyphosphate analysis must be adopted as a routine method for fish assessment in order to avoid fraud, mainly in imported products, which are more prone to polyphosphate addition due to lengthy transportation.

### Ammonia and pH

Ammonia and pH results are presented in Table 2. The increase in pH values indicates an accumulation of alkaline compounds which are produced during the degradation process and, in general, present satisfactory correlation with other parameters that are considered important indicators of fish quality (Monteiro et al. 2010; Islami et al. 2015). Moreover, the pH parameters may indicate the presence of additives such as polyphosphate (Karl et al. 2010; Carneiro et al. 2013b). In the same samples wherein polyphosphate was detected >2 g 100 g^−1^ (30% of total samples), the pH values were >7.00, suggesting that the use of water‐binding additives promotes an increased pH. Similar behavior between pH values and the presence of polyphosphate was observed by Karl et al. (2010) in *Pangasius* fillets.

With the exception of samples with an excessive addition of polyphosphate amounts, our results demonstrate pH values ranging from 5.88 to 6.18. Noseda et al. (2012) observed initial pH values of 6.63 in *Pangasius* fillets. Islami et al. (2015) reported pH values between 6.88–7.07 in *Pangasius* fillets stored in ice for 21 days. Nayak et al. (2015) demonstrated pH values ranging from 6.54 to 7.27 after 12 days of refrigerated storage (6 ± 2°C) of *Pangasius hypophthalmus*. The pH values vary greatly in the literature for *Pangasius hypophthalmus*, probably because this parameter depends on catching, processing, and storing methods as well as the use of additives (Esaiassen et al. 2004; Aursand et al. 2009; Karl et al. 2010; Carneiro et al. 2013b). Therefore, pH values are usually interpreted in association with other quality parameters (Monteiro et al. 2010; Islami et al. 2015).

Regarding ammonia, this compound is produced mainly by oxidative deamination of protein components from fish muscle and by bacterial deamination of amino acids during storage. In addition, ammonia is the alkaline compound more abundant at the beginning of the degradative process (Debevere and Voets 1973) and can markedly increase during storage time of fish species; hence it can be an adequate quality indicator for this food matrix (Rodrigues et al. 2013). On this matter, studies focus on water quality in order to verify possible problems in fish production (Lefevre et al. 2011). There are no reports concerning quantitative assessments of ammonia in fish meat, making it impossible to compare our results with other studies. Nevertheless, in general, the present study demonstrated ammonia concentrations ranging from 0.12 to 0.34 *μ*g NH_3_ g^−1^. Discrepant values (1.87 and 1.94 *μ*g NH_3_ g^−1^) were observed in 20% of the total samples. The presence of ammonia is related to microbial growth and the microbial quality of *Pangasius* fillets depends on some factors related to the aquaculture system such as the quality of the water wherein fish live, fish processing (from raw materials to final products), transport and storage (Orban et al. 2008; Tong Thi et al. 2013). Our findings indicate that 80% of the *Pangasius* fillets from Vietnam sold in the Brazilian market had already initiated the deterioration process whereas 20% of the samples demonstrated an advanced stage of degradation. Therefore, it suggests paying greater attention to ammonia monitoring in *Pangasius* fillets, mainly due to their neurotoxic effects on humans (Thrane et al. 2013).

### Biogenic amines

Biogenic amine results are exhibited in Table 3. Among all BAs analyzed, histamine is the only one regulated by governmental authorities as a result of severe toxicological symptoms when ingested in large quantities (Muñoz‐Atienza et al. 2011). According to European legislation, the histamine limit is 200 mg kg^−1^ for fresh fish (European Commission 2005) whereas the Food and Drug Administration allows 500 mg kg^−1^ (FDA 1998). The Brazilian legislation recommends 100 mg kg^−1^ as the maximum level of histamine in the muscle of fish species from the *Scombridae*,* Scombresocidae*,* Clupeidae*, and *Coryphaenidae* families (Brazil 1997). Histamine was registered only in 20% of the samples, which was much lower than the values recommended by different government regulations, probably due to low histamine levels in this fish species (Tao et al. 2011).

**Table 3:** Biogenic amines in *Pangasius hypophthalmus* fillets from Vietnam marketed in Brazil

Samples	Biogenic amines (mg kg^−1^)				
	Putrescine	Cadaverine	Histamine	Spermine	Spermidine
1	0.64 ± 0.03	0.09 ± 0.00	ND	0.68 ± 0.00	1.59 ± 0.01
2	0.70 ± 0.02	0.77 ± 0.03	ND	0.80 ± 0.00	1.49 ± 0.01
3	0.79 ± 0.05	0.36 ± 0.02	0.02 ± 0.00	0.65 ± 0.02	1.74 ± 0.01
4	0.63 ± 0.02	0.25 ± 0.02	ND	0.61 ± 0.01	1.80 ± 0.01
5	0.84 ± 0.02	0.88 ± 0.02	0.01 ± 0.00	0.03 ± 0.00	1.67 ± 0.01
6	0.79 ± 0.01	1.38 ± 0.02	ND	0.49 ± 0.01	1.49 ± 0.01
7	1.20 ± 0.02	1.45 ± 0.01	ND	0.73 ± 0.01	1.70 ± 0.01
8	0.83 ± 0.04	1.19 ± 0.01	ND	2.98 ± 0.01	4.41 ± 0.01
9	0.58 ± 0.02	0.35 ± 0.02	ND	0.66 ± 0.01	1.67 ± 0.01
10	0.65 ± 0.04	0.66 ± 0.00	ND	0.64 ± 0.01	0.15 ± 0.01

ND, not detectable.

Values were expressed as mean ± standard deviation.

Putrescine, cadaverine, spermidine, and spermine were detected in all samples analyzed. Despite large lysine and arginine contents in *Pangasius hypophthalmus* fillets (Phumee et al. 2011), putrescine, cadaverine, spermidine and spermine concentrations were very low (<3 mg kg^−1^) in our study. Nevertheless, both the presence of putrescine and cadaverine is commonly associated with hygiene conditions, indicating a possible contamination, mainly by gram negative bacteria from the *Enterobacteriaceae* family and *Pseudomonas* genus (Zhai et al. 2012). Moreover, these BAs have been applied as the quality index for freshwater fish (Cunha et al. 2013; Rodrigues et al. 2013). Putrescine and cadaverine can potentiate the toxic effects of histamine and tyramine just as putrescine, spermine, spermidine, and cadaverine may react with nitrates resulting in nitrosamines, which are carcinogenic compounds (Juneja and Sofos 2010).

The BA concentrations can be influenced by storage conditions such as time, temperature, and type of packaging (Krízek et al. 2004). Moreover, season and location variation contribute to different BAs amounts (Gomaa et al. 2011). Despite this, low concentrations of individual BAs were previously reported in several fish species including *Pangasius hypophthalmus* fillets (Zhai et al. 2012; Bunka et al. 2013; Kulawik et al. 2015), which agrees with our findings.

### Lipid oxidation

MDA results are exhibited in Table 2. MDA are secondary compounds from lipid oxidation and, therefore, are commonly used as indicators of oxidative rancidity in fish (Palmeira et al. 2014). MDA determination is of paramount importance due to its ability to negatively influence the sensory properties of foods and human health (Chaijan 2008; Duthie et al. 2013; Zaki et al. 2014). There are no limits established by legislation for MDA values in fish or their derived products. Nevertheless, Connell (1995) reported maximum MDA values of 1–2 mg MDA kg^−1^ of fish flesh. Monteiro et al. (2012) suggested a limit of 1.27 mg MDA kg^−1^ in refrigerated tilapia fillets.

Regarding our MDA results, most values range from 1.21–3.58 mg MDA kg^−1^, however, discrepant MDA values were observed in 20% of the samples (7.44–7.88 mg MDA kg^−1^). When compared to maximum MDA values described by Connell (1995) and Monteiro et al. (2012), 50% and 90% of the samples presented MDA values above permitted limits, respectively. Kulawik et al. (2015) did not detect MDA in frozen *Pangasius* fillets exported to Poland, indicating a satisfactory quality in terms of oxidative rancidity. On the other hand, the same authors observed MDA values between 0.15–0.27 mg MDA kg^−1^ in frozen *Pangasius* fillets exported to Germany and Ukraine (Kulawik et al. 2015). Similar results were reported by Shariat et al. (2013) in fresh *Pangasius* fillets from Malaysia. Our findings indicate that 100% of the samples presented expressive initial values of MDA, suggesting flaws in the fish removal method, handling, processing, and mainly transport and/or storage which can accelerate lipid oxidation. Oxidative rancidity can occur even in low‐fat fishes, depending on the fatty acid composition. It can also occur during refrigerated and frozen storage, which are the most common conservation methods used for fishes conforming to previous reports in literature (Monteiro et al. 2012; Indergard et al. 2014; Karlsdottir et al. 2014; Qiu et al. 2014).

Under frozen temperatures (−5.15 to −18.15°C), there is a decrease in activity of spoilage microorganisms and enzymes present in fish products, delaying the formation of metabolites from protein degradation (Bazarra et al. 2015). However, low temperatures do not prevent lipid oxidation due to the action of endogenous lipoxygenases even under frozen conditions (Karlsdottir et al. 2014). This can explain our findings regarding metabolites resulting from the deterioration process in which lipid changes were more pronounced than protein modifications. In addition, the high MDA values found in our study can be attributed to long transportation and/or storage periods of the *Pangasius* fillets under frozen conditions, taking into account that the Brazilian markets are further in distance compared to European markets.

### Mercury

Among harmful environmental pollutants, mercury (Hg) is one of the most important due to its toxicity in both fish species and humans (Minh 2015). In water, mercuric ions (Hg^2+^) are present in great amounts, whereas in marine organisms, the predominant molecular form is methylmercury (MeHg), which is formed by biomethylation (microorganism action) in aquatic environments. In addition, organic forms such as MeHg exhibit higher toxicity than inorganic species (Ciardullo et al. 2008). The Food and Agriculture Organization and the World Health Organization recommend a limit of 0.5 mg kg^−1^ and 1 mg kg^−1^ in nonpredatory and predatory fishes, respectively. They further recommend the limit of 0.2 mg kg^−1^ for risk groups such as pregnant women, individuals under 15 years or frequent fish consumers (FAO/WHO 2010). *Pangasius* is an omnivorous species that mainly consumes rice bran, soy, and fish by‐products (Hung et al. 2004).

Our results demonstrated that 50% of the samples exhibited Hg values above the recommended limit (0.51–1.31 mg kg^−1^) (Table 2). Safety Hg levels (<0.5 mg kg^−1^) were reported by Orban et al. (2008), Szlinder‐Richert et al. (2011), Mok et al. (2012), Pal et al. (2012), Ruiz‐de‐Cenzano et al. (2013) and Kulawik et al. (2015) in *Pangasius* fillets from Vietnam and southern or southeast Asia. Nevertheless, in line with our findings, 50% of *Pangasius* fillets from Vietnam marketed in Italy demonstrated Hg concentrations >0.5 mg kg^−1^ (Ferrantelli et al. 2012). These results demonstrate the need for control measures in order to improve water quality where *Pangasius* are farmed, as well as to better monitor the quality of these fish species during importation in order to check for the contamination risks of fish consumption.

Regarding the relevance of the chemical parameters investigated and the findings found in the present experiment, it suggested a potential risk in this matrix. Therefore, efforts should be carried out for the establishment of a strong control system to ensure the health of consumers by way of monitoring the chemical quality of *Pangasius* fillets.

## Conclusions

This study confirms the presence of detrimental compounds in Vietnamese *Pangasius hypophthalmus* fillets. Hg and MDA were the main chemical hazardous compounds observed in more than 50% of the studied samples. Therefore, there is a need for quality control points in the *Pangasius* production chain in order to promote product standardization and produce a safer food supply chain which is in global demand (consumers and marketers).

## Conflict of Interest

None declared.
